# Supplementary material for: The Mechanism of SARS-CoV-2 Nucleocapsid Protein Recognition by the Human 14-3-3 Proteins
Source: J Mol Biol. 2021 Apr 16;433(8):166875. doi: 10.1016/j.jmb.2021.166875 (PMC7863765; doi:10.1016/j.jmb.2021.166875)

**Fragmentation spectra (ETD or HCD) of phosphopeptides from NCAP_SARS2 Nucleoprotein (P0DTC9) corresponding to the potential 14-3-3-binding sites (chymotrypsin or trypsin and LC-MS/MS)**

**A)**


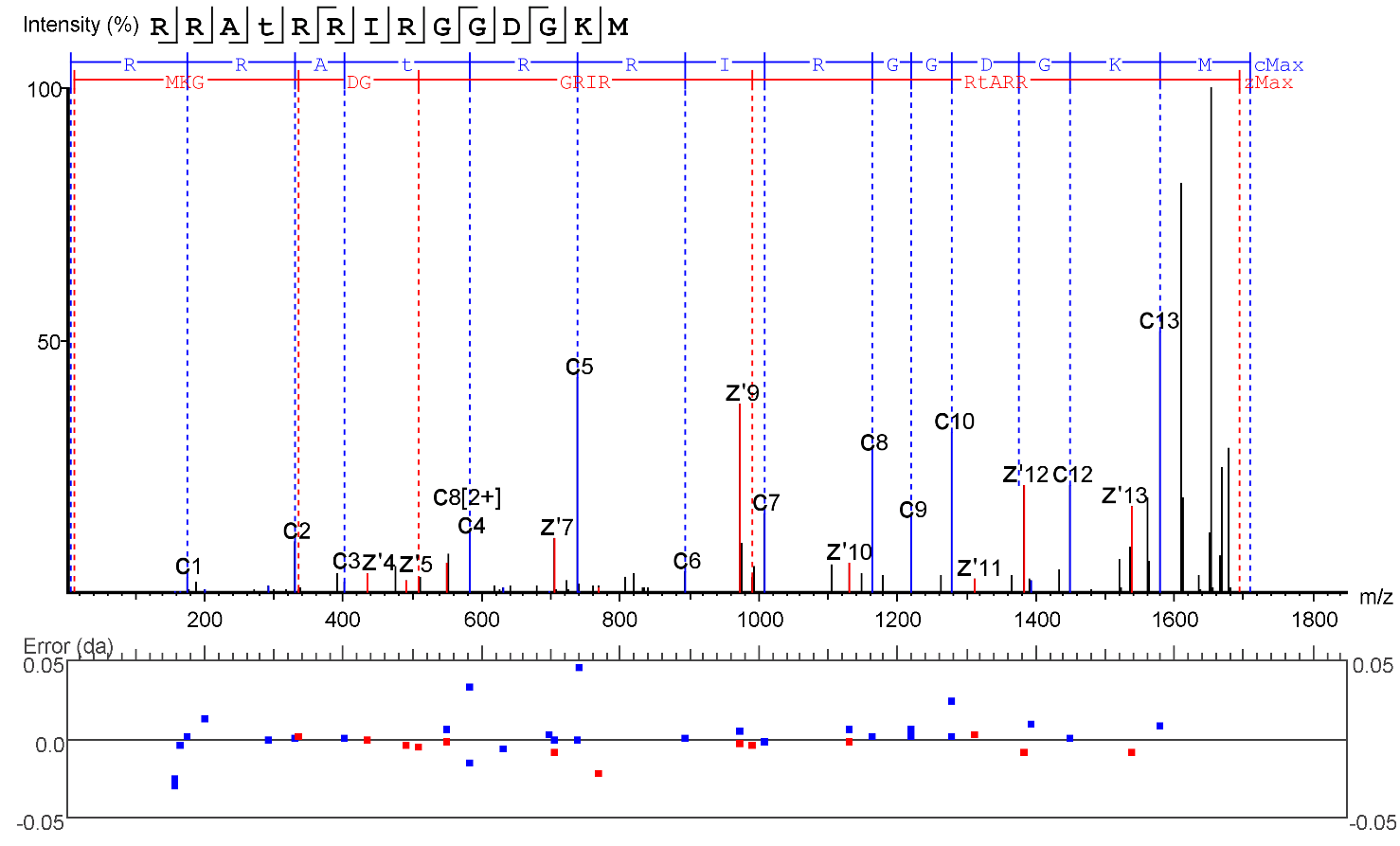


**B)**
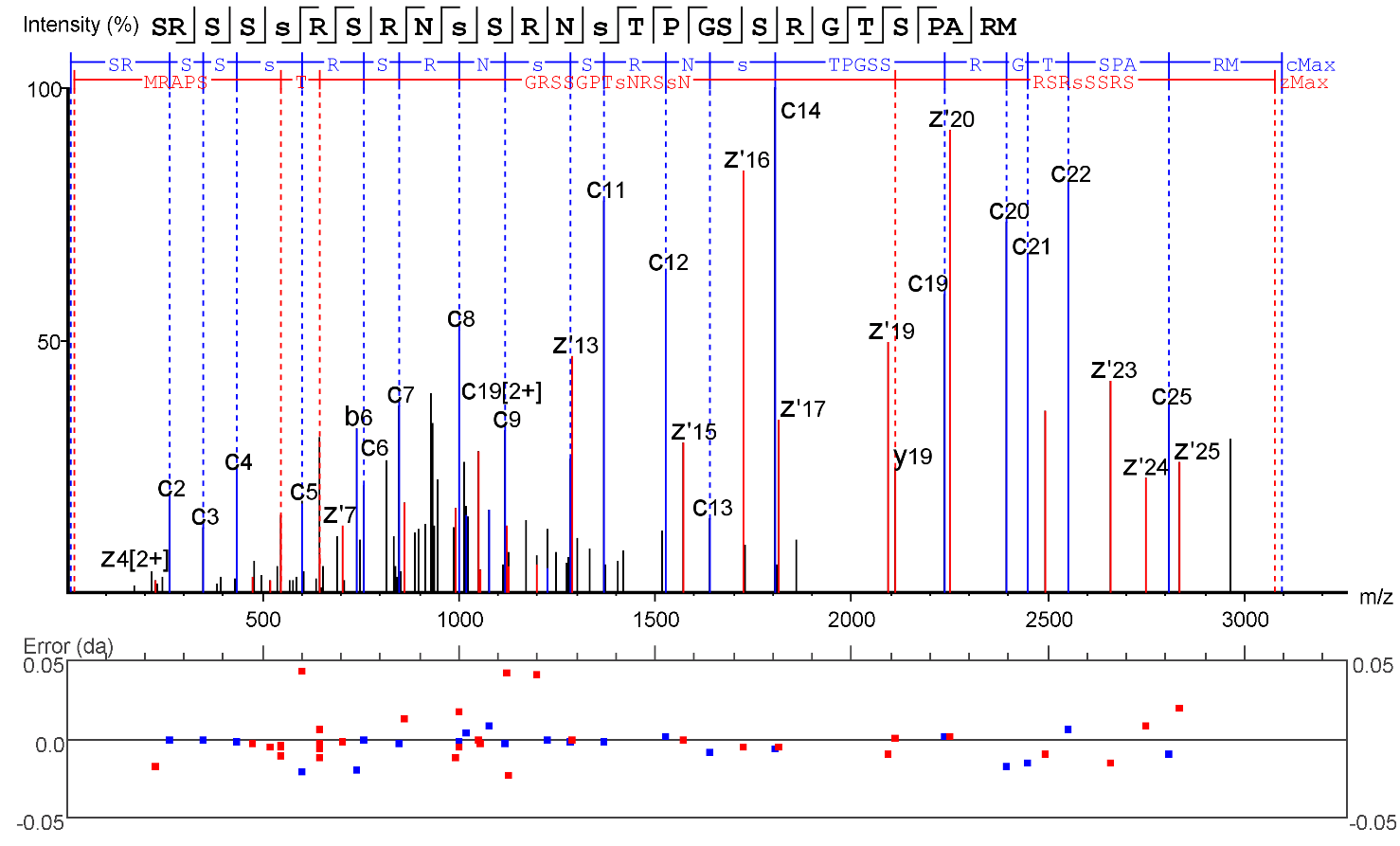


**C)**


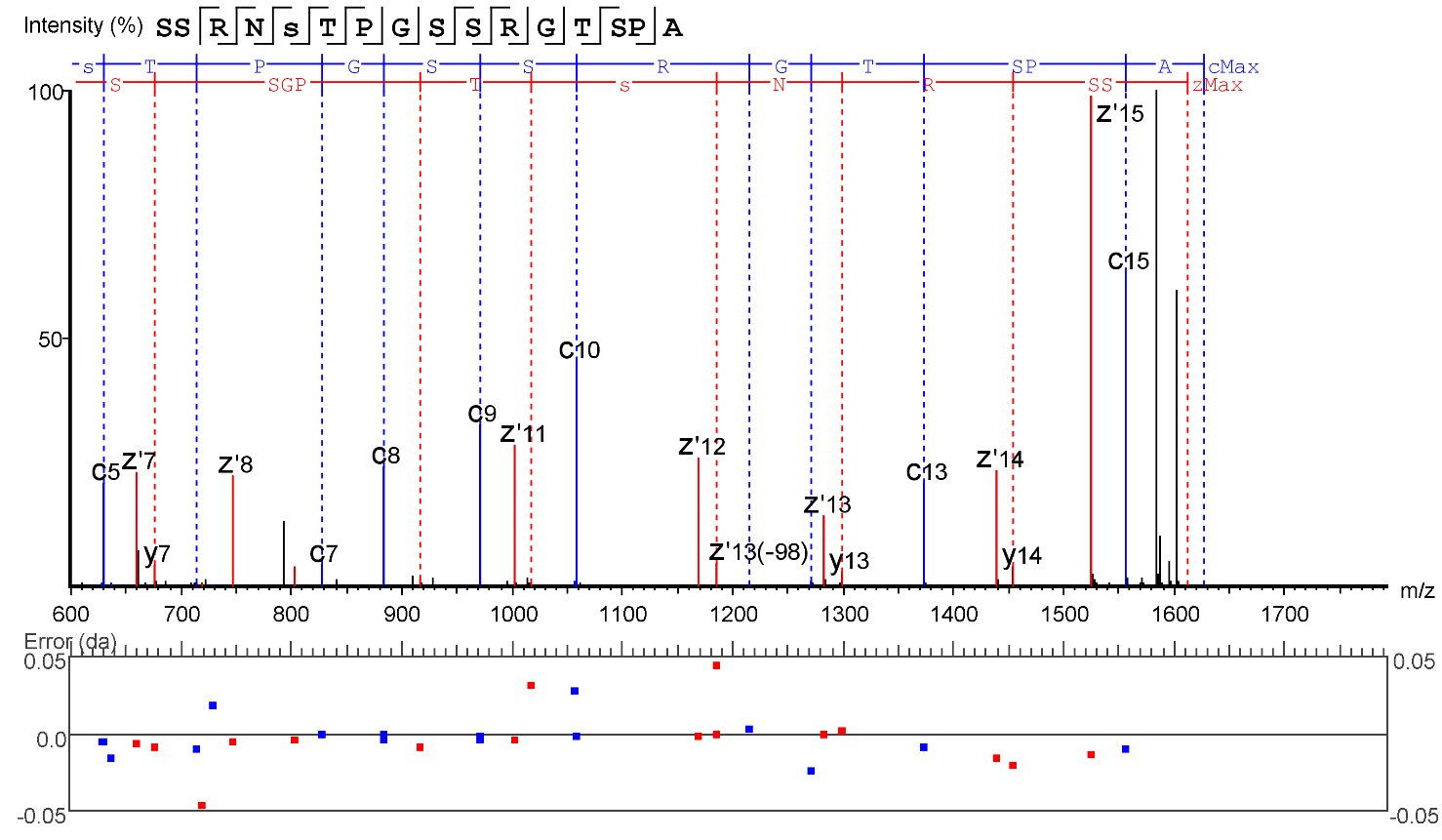


**D)**


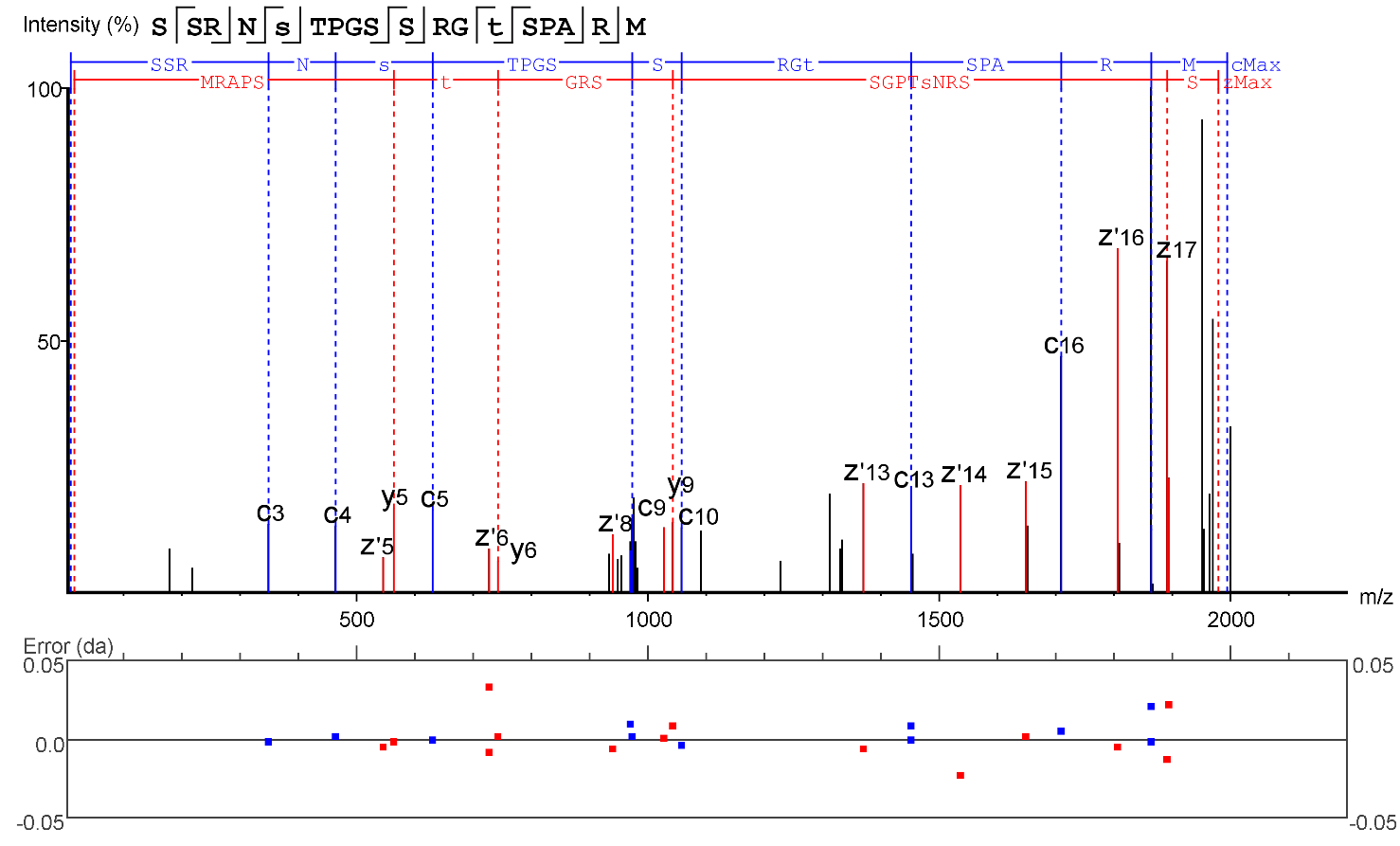


**E)**


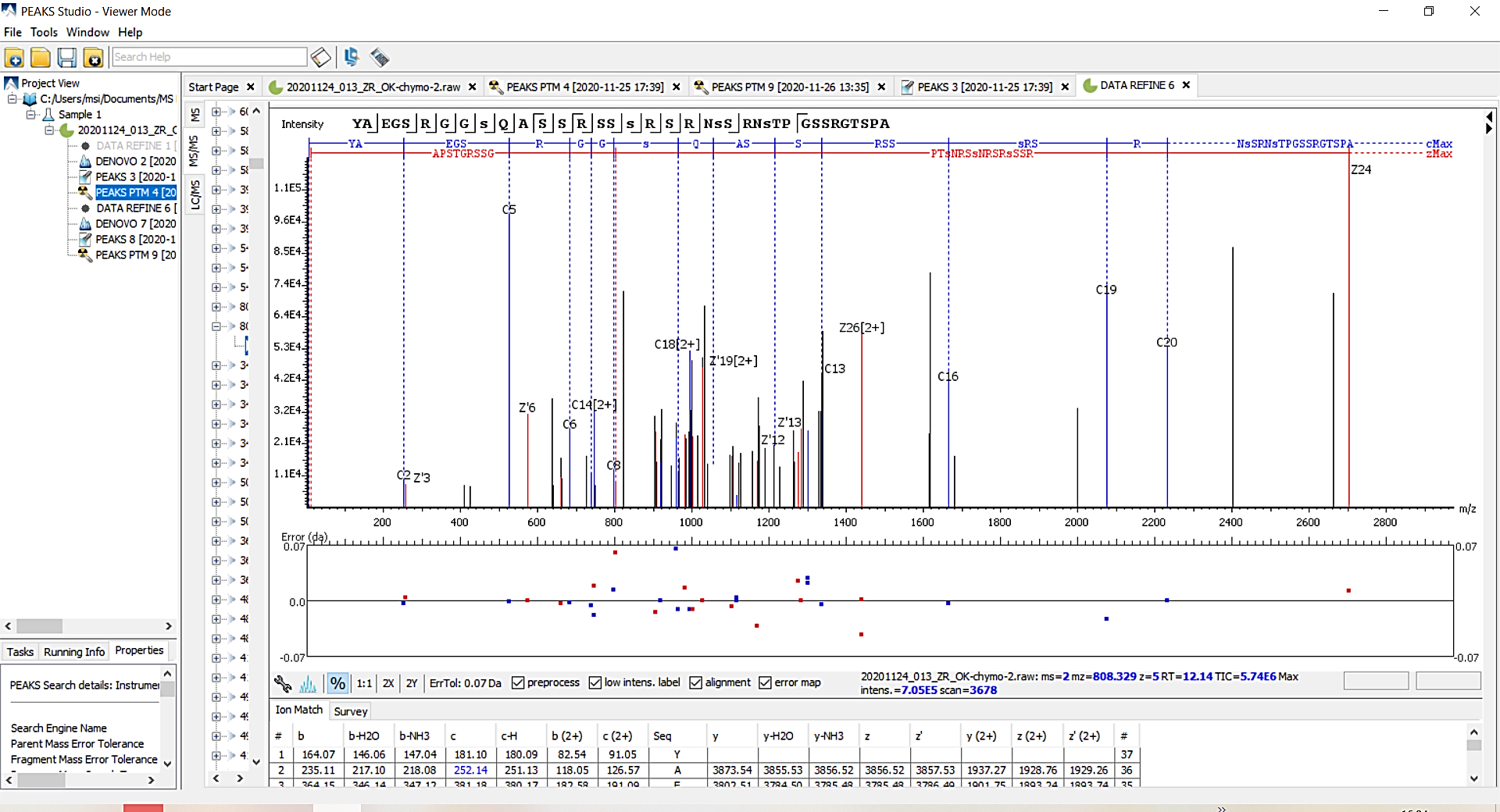


**F)**TAt_⎦_ K_⎦_ A_⎦_ Y_⎦_ N_⎦_ V_⎦_ TQAFGR


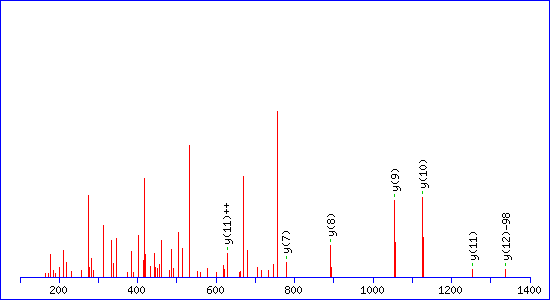


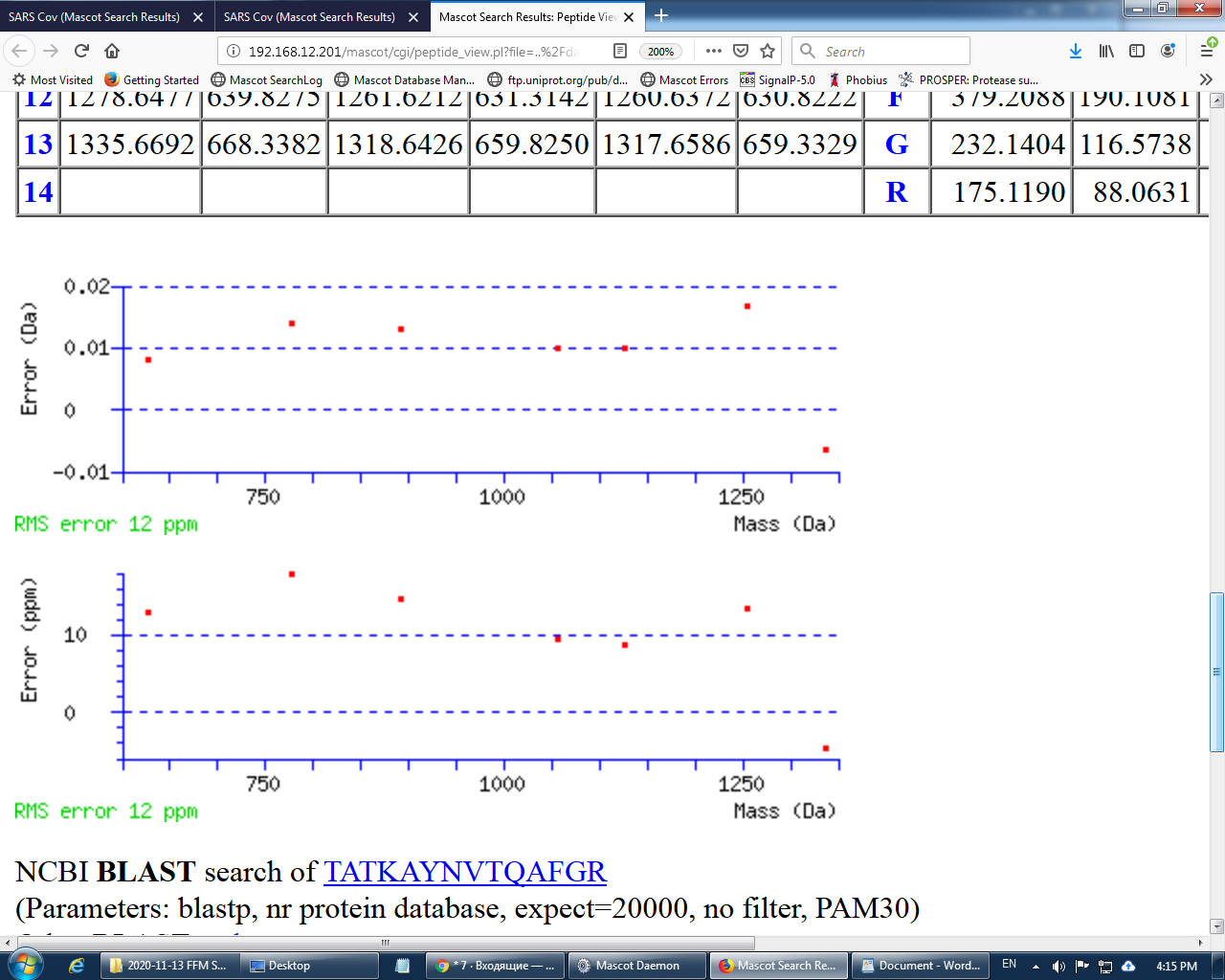

Supplement: Supplementary data 4 [file mmc4.docx]
