# Supplementary material for: The Mechanism of SARS-CoV-2 Nucleocapsid Protein Recognition by the Human 14-3-3 Proteins
Source: J Mol Biol. 2021 Apr 16;433(8):166875. doi: 10.1016/j.jmb.2021.166875 (PMC7863765; doi:10.1016/j.jmb.2021.166875)
Supplement: Supplementary data 5 [file mmc5.docx]

**Phospho mapping of NCAP_SARS2 Nucleoprotein (P0DTC9) by LC-MS/MS.** The data is a combined result of 4 independent LC-MS/MS experiments. A protein sample was digested either with trypsin or with chymotrypsin and MS data were searched against concatenated SARS-CoV-2 and general contaminants database. For Mascot (Matrix Science) search the parameters were: Enzyme – Trypsin with 1 miscleavage allowed or no enzyme; Fixed Modifications – Methylthio(C); Variable modifications – Oxidation(M), Phospho(ST), Phospho(Y); Peptide tolerance for MS – 5 ppm; MS/MS tolerance- 0.2 Da; Decoy algorithm was used to estimate FDR. For PEAKS (PEAKS Studio Xpro, Bioinformatics Solutions Inc.) PTM matching the parameters of a analysis were: MS tolerance - 10 ppm, MSMS – 0.05 Da; Fixed modifications – Methylthio(C); Variable modifications – Phospho(STY), Oxidation(M), Ammonia-loss(N), Deamidation(NQ). Peptides with Mascot peptide score ≤20 and PEAKS –l0lgP ≤20 were filtered out. To consider that amino acid was phosphorylated it should appear at least two times ether in one search or in two independent searches. One times appearing phosphosites were not taken into analysis.

10 20 30 40 50 60

MSDNGPQNQR NAPRI**T**FGGP SD**ST**GSNQNG ERSGAR**S**KQR RPQGLPNNTA SWFTALTQHG

70 80 90 100 110 120

KEDLKFPRGQ GVPINTNSSP DDQIGYYRRA **T**RRIRGGDGK MKDLSPRWYF YYLGTGPEAG

130 140 150 160 170 180

LPYGANKDGI IWVATEGALN TPKDHIGTRN PANNAAIVLQ LPQGTTLPKG FYAEGSRGG**S**

190 200 210 220 230 240

QASSR**SSS**R**S** RN**SS**RN**ST**PG **SS**RG**T**SPARM AGNGGDAALA LLLLDRLNQL ESKM**S**GKGQQ

250 260 270 280 290 300

QQGQTVTKKS AAEASKKPRQ KRTA**T**KAYNV TQAFGRRGPE QTQGNFGDQE LIRQGTDYKH

310 320 330 340 350 360

WPQIAQFAPS ASAFFGMSRI GMEVTPSGTW LTYTGAIKLD DKDPNFKDQV ILLNKHIDAY

370 380 390 400 410 420

KTFPPTEPKK DKKKKADETQ ALPQRQKKQQ **T**V**T**LLPAADL DDF**S**KQLQQ**S** MS**S**ADSTQA
